# 631. Cost-effective diagnostic algorithms for Ugandan patients with solid tumors who develop chemotherapy-associated febrile illness

**DOI:** 10.1093/ofid/ofae631.196

**Published:** 2025-01-29

**Authors:** Elizabeth Gulleen, Immaculate Mbrusha, Dennis Mubiru, Bernadette Pedun, Abrahams Omoding, Christopher Moore, Warren Phipps, Radha Rajasingham

**Affiliations:** University of Minnesota, Minneapolis, MN; Uganda Cancer Institute, Kampala, Kampala, Uganda; Uganda Cancer Institute, Kampala, Kampala, Uganda; Uganda Cancer Institue, Kampala, Kampala, Uganda; Uganda Cancer Institute, Kampala, Kampala, Uganda; University of Virginia, Charlottesville, Virginia; Fred Hutchinson Cancer Research Center; University of Washington, Seattle, WA; University of Minnesota, Minneapolis, MN

## Abstract

**Background:**

Microbiology diagnostic tests are often prohibitively expensive in resource-limited settings. Cost-effective diagnostic algorithms could potentially guide diagnostic evaluation for chemotherapy-associated febrile illness in resource-limited settings where comprehensive testing is not possible. This is particularly true in sub-Saharan Africa, where the high prevalence of HIV and tuberculosis (TB) place patients at increased risk of developing opportunistic infections that do not frequently occur among patients receiving chemotherapy in the United States or Europe.

**Methods:**

We created a decision analytic model to evaluate the costs, diagnostic yield, and cost-effectiveness of diagnostic algorithms for adult inpatients with solid tumors who developed fever within 30 days of receiving chemotherapy in Uganda. Since Uganda has a high prevalence of HIV and TB, diagnostics included serum cryptococcal antigen (CrAg) lateral flow assay, Alere TB urinary lipoarabinomannan (LAM), malaria rapid testing, and aerobic blood cultures. We considered the following testing algorithms: 1) a comprehensive approach where all tests were performed, and 2) various stepwise algorithms where tests were performed in series and stopped testing with the first positive result. We obtained prevalence data from a cohort of 100 patients with solid tumors who were admitted to the Uganda Cancer Institute (Kampala, Uganda) with fever. Test performance was taken from the published literature.

**Results:**

A comprehensive diagnostic testing strategy yielded 64% of correct diagnoses at a cost of $97.85 per correct diagnosis. Of the stepwise strategies, testing CrAg first yielded the cheapest strategy, at a cost of $85.49 per correct diagnosis, and the highest number of appropriate diagnoses (59%). The incremental cost-effectiveness ratio was $234 per additional correct diagnosis for the comprehensive over this sequential strategy.

**Conclusion:**

Comprehensive diagnostic testing is optimal for patients who experience post-chemotherapy febrile illness in resource-limited settings. If this strategy is not available, stepwise testing with CrAg then TB LAM is the optimal strategy to maximize correct diagnosis and minimize costs.

**Disclosures:**

**All Authors**: No reported disclosures

